# Formulation of HBs antigen in Montanide ISA266 shows superiority to commercial HBsAg vaccine in the induction of humoral immune responses 

**Published:** 2019

**Authors:** Mohammad Ali Savoji, Setareh Haghighat, Mina Mirzaee, Bahareh Golkaran, Rayhaneh Mirzaee, Behzad Esfandiari, Mehdi Mahdavi

**Affiliations:** 1 *Department of Microbiology, Faculty of Advanced Sciences & Technology, Tehran Medical Sciences, Islamic Azad University, Tehran, Iran*; 2 *Department of Epidemiology and Biostatistics, Pasteur Institute of Iran, Tehran, Iran*; 3 *Department of Laboratory Animal Science, Pasteur Institute of Iran, Karaj, Iran*; 4 *Recombinant Vaccine Research Center, Tehran University of Medical Sciences, Tehran, Iran *; 5 *Immunotherapy Group, The Institute of Pharmaceutical Sciences (TIPS), Tehran University of Medical Sciences, Tehran, Iran *; 6 *Department of Immunology, Pasteur Institute of Iran, Tehran, Iran *

**Keywords:** Adjuvant, Hepatitis B vaccine, Montanide ISA 266, Immune response

## Abstract

**Aim:**

In the present study, a new formulation of HBsAg vaccine was developed and compared with a commercial peer.

**Background:**

Vaccination of hepatitis B infection has been an unavoidable affair since the 1980s, though it has numerous limitations such as inefficacy in the induction of cellular immune responses. To address these limitations, research on novel formulations is necessary to develop a superior formulation with the potency of induction of both cellular and humoral immune responses.

**Methods:**

HBsAg was formulated in oil-in-water adjuvant Montanide ISA-266 (5 µg/dose) using homogenizer. Balb/C mice were then immunized three times at days 0, 14, and 28 with HBsAg/Montanide ISA-266 or HBsAg/alum with proper control groups. Two weeks after the last immunization, immunological parameters including IL-2, IL-4, TNF-α, IFN-γ, total IgG and IgG1/IgG2a isotypes were assessed by ELISA.

**Results:**

The results demonstrated that the formulation of HBsAg with Montanide ISA-266 enhanced humoral immune responses versus the commercial vaccine and control groups. No significant difference in terms of Th1 pattern was found between HBsAg/Montanide ISA-266 and the commercial vaccine.

**Conclusion:**

Formulation of HBsAg with an oil-based adjuvant may be useful for the induction of a more potent humoral immune response compared to the commercially available HBV vaccine.

## Introduction

 Hepatitis B virus (HBV) is a member of the hepadnaviridae family (1-4). HBV causes infection in humans that may result in acute or chronic hepatitis, liver cirrhosis, and hepatocellular carcinoma (5, 6). The infection may occur in early childhood or older ages (7). The prevention of Hepatitis B infection mainly relies on vaccination. Commercial hepatitis B vaccine is based on HBsAg that formulated in alum adjuvant. After three times immunization, this vaccine leads to a humoral immune response and antibody production at a protective level that is higher than 100 IU/ml (8). Although this vaccination strategy has mead it possible to reduce the rate of the infection, some vaccine recipients produce less than ten mIU/ml anti HBsAg titer after immunization and thus coincided as non-responders (9-13). HBs vaccine is usually highly immunogenic. However, certain factors have been associated with low responsiveness to HBsAg vaccine, including genetic background, obesity, hemodialysis, age, and tobacco consumption (14). To address this problem, adjuvants have been used for increasing vaccine potency such as depot effect and induction of T lymphocytes. Alum is a typical adjuvant that has been widely used in human vaccines to improve humoral immune responses. Besides, adsorption of antigens onto alum results in the high local concentration of antigens and improves antigen uptake by antigen-presenting cells (APCs). Alum components promote immune responses via stimulation of dendritic cells and complement activation. Although alum through induction of chemokines leads to increased immune cells recruitment. It is incapable of inducing cellular immunity (17-18). Thus, a more a potent adjuvant or optimization of alum adjuvant is required to achieve potent cellular immune response. Oil based adjuvants have been mainly shown to increase antibody production. However, in particular situations, they are also able to activate T cells, though at the cost of a possible increased inflammatory reaction at the injection site (19). 

Montanide family adjuvant is a water-in-oil adjuvant with the capacity of inducing a robust immune response. Montanide adjuvants work through forming a liposome surrounding the antigen and protect the antigen from protease degradation. In this way, the antigen is transferred from the injection site to regional lymph nodes without denaturation while the three-dimensional structure of antigen is preserved. Several Montanide adjuvants such as Montanide ISA 70, 51 VG, 720, 206, 266 are commercially available. Based on their type, these adjuvants may either promote higher levels of the humoral immune response or induce a combination of cellular and humoral immune responses. Montanide ISA 266 adjuvant causes a gradual antigen release which can stimulate both cellular and humoral immune responses (20). Studies have shown that water-in-oil adjuvants were able to enhance follicular helper T cells (Tfh) associated responses and stimulate T cell function. Montanide may also develop a more potent humoral immunity via interaction with Tfh cells in the germinal center to trigger antibody production (21-23). In this paper, we hypothesized that formulating HBsAg in Montanide ISA 266 as a water-in-oil adjuvant may lead to enhanced cellular and humoral immune responses in comparison to the commercial HBsAg vaccine. 

## Methods

**HBV vaccines and HBsAg**

Commercial HBsAg vaccine (formulated in alum) and purified HBsAg was provided as in-kind support by the Department of Hepatitis B Vaccine Production, Production & Research Complex, Pasteur Institute of Iran (Karaj, Iran). Formulation of HBsAg in Montanide ISA 266 (SEPPIC, France) was carried out using a standard protocol. In brief, five µg of HBsAg was admixed with Montanide ISA 266 (a ratio of 30/70) and the vigorous vortex was done for 5 minutes to achieve a creamy sample. The sample was then homogenized with a homogenizer three times for 60 seconds, with 60 seconds intervals to obtain a homogenous suspension (15). The homogeneity was confirmed by microscopic examination. This formulation, containing 5 µg of HBsAg in each 100 µl of vaccine, was used for immunization.

**Experimental mice and immunization protocol**

Mice, including a six-to-eight week old female inbred BALB/c (n=30), were purchased from Pasteur Institute of Iran (Karaj, Iran). The mice were acclimatized for seven days before initiation of the experiments with free access to food and water in a standard condition including equal light/dark cycles and temperature ranging between 20 and 22C. The Animal Care Protocol of the Pasteur Institute of Iran was followed during the experiment. Experimental female BALB/c mice were divided into five groups (n=4-6). Group1 was administrated a commercial HBsAg vaccine (5 µg) formulated in alum and group 2 received the vaccine formulated in Montanide ISA266 adjuvant. Groups 3-5 served as control groups and received PBS, alum, or Montanide ISA 266, accordingly. The experimental mice received three subcutaneous injections with two-week intervals on days 0, 14, and 28. 

**IFN-γ, IL-2, TNF-α, and IL-4 cytokines assay**

Two weeks after the last immunization, animals were euthanized, and spleen cell suspensions were prepared by mechanical dissociation of spleens in cold PBS containing 2% fetal bovine serum (FBS) and antibiotics under sterile condition. Red blood cells were then lysed by adding 5 mL of lysis buffer (NH_4_Cl+Tris Base) to cell pellets, and single-cell suspensions were prepared and adjusted to 3×10^6^ cells/ml in complete RPMI-1640. One milliliter of cell suspension containing 3×10^6^ cells was cultured in each well of 24-well plate and stimulated with 5μg/ml of HBsAg for 60 hrs at 37°C in 5% CO2. The supernatants were then collected and used for assessment of IFN-γ, IL-2, TNF-α and IL-4 cytokines in parallel to recombinant cytokines as standards (at concentrations of 1000,500,250,125,62.5,31.25,15.6 and 7.8 pg/ml) using commercial ELISA Kits (Mabtech, Sweden) according to the manufacturer’s instruction. The concentration of each cytokine was calculated according to its standard curve and reported as pg/ml. IFN-γ/IL-4 ratio for individual mice was also calculated by dividing the concentrations of IFN-γ by IL-4 in each mouse.

**Specific total IgG responses and IgG1/IgG2a isotypes by ELISA**

Specific total IgG antibodies after first, second, and third immunization course were evaluated using an optimized indirect ELISA. Briefly, wells of 96-well ELISA Maxisorp plates (Nunc, Naperville, IL) were coated with 100µl aliquots of HBsAg (5µg/ml) in PBS and incubated at 4˚C overnight. The wells were then washed three times with PBS containing 0.05% Tween 20 (washing buffer) and blocked for 1hr at 37˚C with 5% skimmed milk in washing buffer (blocking buffer). Serial dilutions of sera in the order of 1/25 to 1/838860800 were prepared in 1% PBS-BSA containing 0.05% Tween 20 (dilution buffer). After washing the wells, 100µl of each dilution was added to wells in duplicates and incubated at 37˚C for 2 hrs. The wells were then washed five times with washing buffer, and 100µl of 1/10000 dilution of anti-mouse conjugated to horseradish peroxidase (HRP, Sigma, USA) in dilution buffer was added and incubated for 2 hrs at 37˚C. The wells were then washed five times with washing buffer, and 100µl of TMB substrate was added and incubated for 30 min in the dark. The reaction was stopped using 100 l of 2N H_2_SO_4_ and color density was measured at A_450/630 _nm with an ELISA plate reader (AWARENESS technology, USA). To detect specific IgG1, IgG2a, goat anti-mouse IgG1, IgG2a secondary antibodies (Sigma, USA) were used according to the manufacturer’s instruction.

**Statistical analysis**

The results of immunoassays are presented as mean±SD of each duplicate or triplicate measurement. The data were analyzed using Graph pad prism V6.01 software. Mann Whitney U test was applied to compare the statistical differences among the experimental groups. P-values less than 0.05 were considered to represent statistically significant differences between experimental groups. 

## Results

**Cytokines measurement**

**Figure 1:**
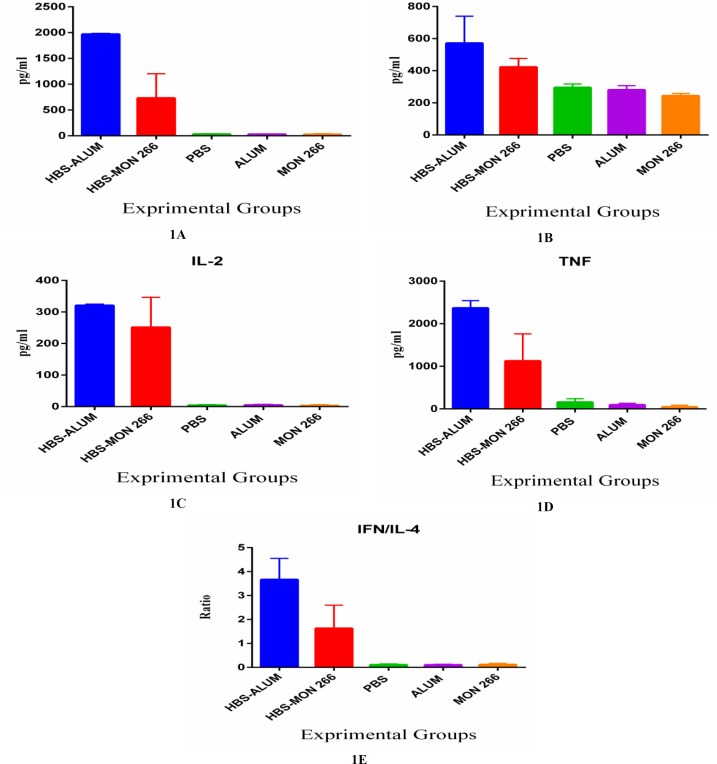
Cytokines responses in experimental groups. IFN-γ (Fig 1a.), IL-4 (Fig 1b.), IL-2 (Fig 1c.), TNF-α (Fig 1d.) and IFN-γ/IL-4 ratio (Fig 1e.) in the experimental groups show that immunization with HBsAg/alum resulted in higher Th1 and Th2 cytokines versus Montanide ISA 266 formulated vaccine. Data are shown as mean ± SD

Results of IFN-γ cytokine measurement showed that both Montanide ISA 266 and alum formulated vaccines increased the level of IFN-γ production versus the control groups (P<0.0001). This increase was significantly more prominent in the HBsAg/alum compared to Montanide ISA 266 formulated vaccine (P<0.0001) (Fig 1a).

Immunization with HBsAg/alum significantly increased IL-4 cytokine versus the control groups (P<0.0003). Immunization with Montanide ISA 266 formulated vaccine increases the level of IL-4 cytokine secretion versus PBS and Montanide ISA 266 control groups (P=0.138 and P=0.451, respectively). HBsAg/alum also significantly increased IL-4 cytokine versus Montanide ISA 266 formulated vaccine (P=0.0321) (Fig 1b).

Results of IL-2 cytokine measurement showed that both Montanide ISA 266 and alum formulated vaccines increased the level of IL-2 compared to the control groups (P<0.0001).

Immunization with HBsAg/alum resulted in significantly more increase in IL-2 production versus HBsAg-Montanide ISA 266 (P=0.0217) (Fig 1c).

TNF-α cytokine assessment in the experimental groups showed that immunization with HBsAg formulated in both Montanide ISA 266 and alum adjuvants significantly increased TNF-α secretion in comparison to the control groups (P<0.0001). Similar to other cytokines, immunization with HBsAg/alum led to a considerably higher level of TNF-α cytokine production versus HBsAg-Montanide ISA 266 (P=0.0001) (Fig 1d).

Comparing IFN-γ/IL-4 ratios among experimental groups indicated that the rate was significantly higher in both Montanide ISA 266 and alum adjuvants groups in comparison to the control groups (P<0.0069). IFN-γ/IL-4 ratio was again markedly higher with HBsAg/alum immunization than HBsAg/Montanide ISA 266 vaccine (P=0.0001) (Fig 1e).

**Specific total IgG**

Specific total IgG after the first immunization with HBsAg/alum at dilutions of 1/25 and 1/50 significantly increased versus PBS control (P<0.0136) and at dilutions of 1/25, 1/50 and 1/100 showed a significant increase versus the alum control group (P<0.0001).

**Figure 2:**
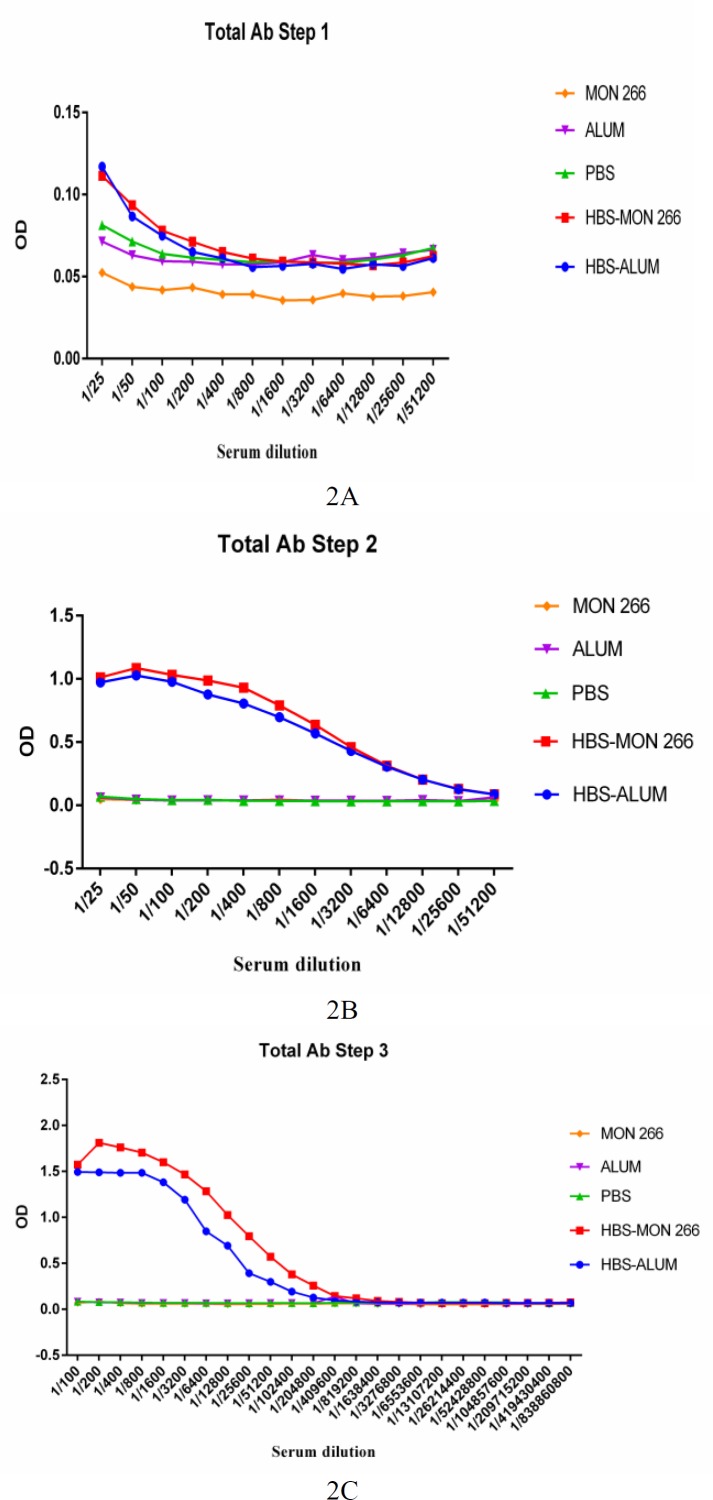
Specific total IgG responses in experimental groups after first (Fig 2a.), second (Fig 2b.) and third (Fig 2c.) immunization rounds. Immunization with both vaccines raised total IgG after the first and second immunization, with no significant difference between them. Following the third immunization, HBsAg formulated in Montanide ISA 266 showed superiority in the induction of total IgG versus the commercial vaccine

 Immunization with HBsAg/Montanide ISA 266 at dilutions of 1/25, 1/50 and 1/100 significantly increased the total IgG versus PBS control group (P<0.0111) and at dilutions of 1/25 to 1/51200 (except 1/6400, P=0.0597) showed a significant increase versus Montanide ISA 266 control (P<0.0376). Immunization with HBsAg/Montanide ISA 266 did not show substantial differences versus HBsAg/alum at any of dilutions (P>0.6526) (Fig 2a).

 Specific total IgG after the second immunization with HBsAg/alum at dilutions of 1/25 to 1/6400 significantly increased total IgG versus PBS and alum control groups (P<0.0067). Immunization with HBsAg/Montanide ISA 266 at dilutions of 1/25 to 1/12800 significantly increased total IgG versus PBS and Montanide ISA 266 control groups (P<0.0317). Again, Immunization with HBsAg/Montanide ISA 266 did not show significant differences versus HBsAg/alum any of dilutions (P>0.6771) (Fig 2b).

Specific total IgG after the third immunization with HBsAg/alum at dilutions of 1/100 to 1/51200 significantly increased total IgG versus PBS and alum control groups (P<0.0001). Immunization with HBsAg/Montanide ISA 266 at dilutions of 1/100 to 1/102400 significantly increased total IgG versus PBS and Montanide ISA 266 control groups (P<0.0011 and P<0.0028, respectively).

 Immunization with HBsAg/Montanide ISA 266 at dilutions of 1/200,1/400 and 1/3200 up to 1/51200 showed a significant increase versus HBsAg/alum group (P<0.0081) (Fig 2c).

**Specific IgG isotypes**

**Figure 3:**
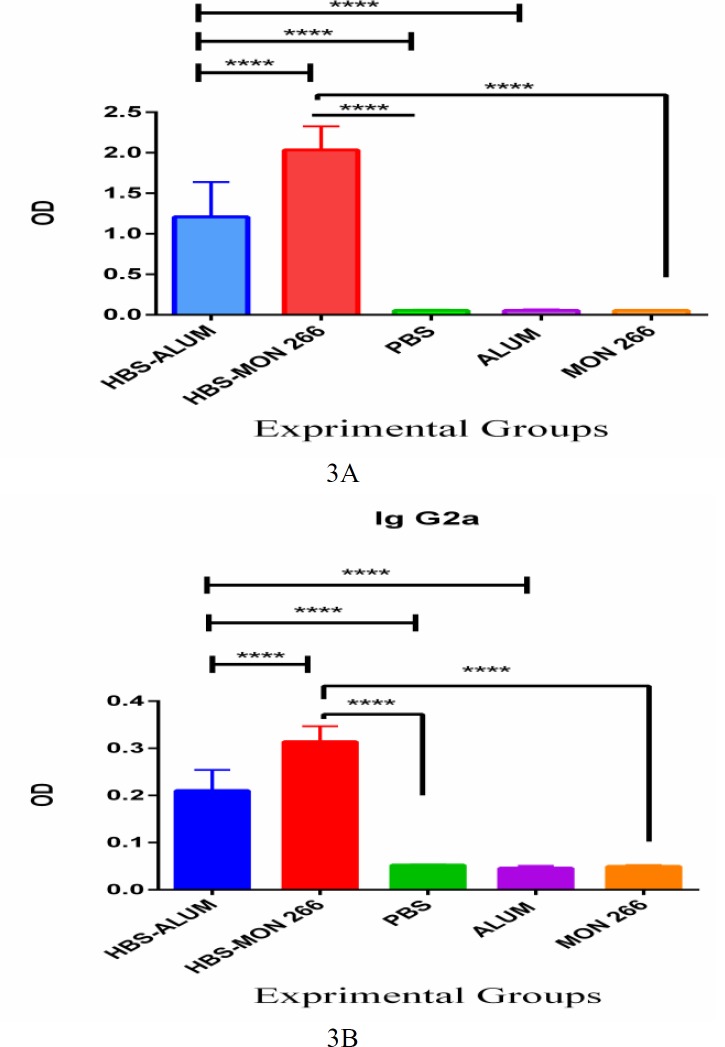
Specific IgG1 (Fig 3a.) and IgG2a (Fig 3b.) antibodies in the experimental groups. Results of particular IgG1/IgG2a shows a higher response in the group that immunized with HBsAg formulated in Montanide ISA 266 versus the commercial vaccine

Immunization with both HBsAg/Montanide ISA 266 and HBsAg/alum increased the level of specific IgG1 versus control groups (P<0.0001). This increase was significantly more prominent with HBsAg/ Montanide ISA 266 immunization versus HBsAg/alum (P=0.0001) (Fig 3a).

Immunization with both HBsAg/Montanide ISA 266 and HBsAg/alum increased the level of specific IgG2a versus control groups (P<0.0001). Similar to IgG1, increase in IgG2a levels was also significantly higher with HBsAg/Montanide ISA 266 immunization versus HBsAg/alum (P=0.0001) (Fig 3b).

## Discussion

 Hepatitis B is currently considered as a life-threatening disease with a high mortality rate in the absence of successful treatment (16). While immunization with the commercial HBsAg vaccine as a preventive measure decreases the incidence of infection in the community, some vaccine recipients (known as non-responders) don’t show enough humoral immune response (12, 16). Herein, we hypothesized that formulation of HBsAg in an oil-based adjuvant might improve humoral and cellular immune responses toward the vaccine. To this end, we developed a new formulation of HBsAg in Montanide ISA 266 adjuvant and assessed its effect on cellular and humoral immune responses. 

 Results of cytokines assays showed that the increase in IFN-γ, IL-2, IL-4, TNF-α, and IFN-γ/IL-4 ratio was higher with the administration of HBsAg/alum compared to HBsAg/Montanide ISA 266 group. This finding suggests that alum was more potent in the induction of Th1/Th2 and inflammatory cytokines than the Montanide ISA 266 based vaccine. Th1 cytokine pattern is essential in controlling viral infection (17). Many studies showed that IL-2, IFN-γ, and TNF-α are involved in the elimination of viral infection through modulating Th1 pattern and inflammatory responses (17, 18). A study by Roohvand et al. showed that HCV formulated with Montanide ISA 720 plus CPG could increase IFN-γ and IL-4 cytokines and CD8+ CTL response (19). This demonstrates the possibility that Montanide can enhance Th1 cytokine production, leading to a reinforced cellular immunity (19).

 In another study, immunization with a recombinant Leishmania vaccine formulated in Montanide ISA 720 and ISA 50V2, resulted in a Th1 pattern immune response in a mouse model (20). Other studies confirmed that various types of Montanide adjuvants are were able to induce cellular immunity via the production of IFN-γ cytokine (21, 22). Jang et al. found that IL-2, IFN-γ, IL-10, and IL-17 were increased by administration of profilin formulated in Montanide ISA 71 as an oil-based adjuvant in chicken with E. maxima infection (23). 

 Here, we achieved a less potent of Th1 pattern using Montanide ISA 266 adjuvant in comparison to the alum adjuvant for HBsAg vaccine. This attenuated efficacy may be due to the nature of our oil adjuvant (24, 25). The behavior of oil adjuvants in the induction of immune responses depends on the nature of fatty acid and type of emulsifier that is employed in the oil adjuvant formulation (24-27). These characteristics are highly variable in different kinds of Montanide ISAs adjuvants (25, 27). 

 Results of the antibody responses after the first and second immunization showed that HBsAg formulated in both alum and Montanide ISA 266 adjuvants are capable of enhancing specific IgG antibody production as compared with control groups. However, no significant differences were observed between alum and Montanide ISA 266 formulated vaccines. However, after the third immunization, Montanide ISA 266 adjuvant resulted in a higher IgG antibody level versus alum adjuvant. Higher IgG response after three immunizations in an oil-based adjuvant shows the potency of this adjuvant for induction of more potent humoral immune responses versus alum-based vaccine. This observed difference may be related to the kinetic of antigen release and a higher frequency of plasma cells in the new formulation group. Also, considering that liposome formation protects antigens from protease and other denaturing agents, these mechanisms may be involved in the more robust antibody responses observed in Montanide formulation group (21, 28). 

 In line with our findings regarding potent induction of humoral immune responses with Montanide formulation, various other studies have also shown that oil-based and Montanide ISAs formulated vaccines were more dominant in the induction of humoral immune responses versus alum based vaccine (29). It is well-known that humoral immune responses can be affected by T cells through cytokines production (30). Results of the present study, however, show that while HBsAg/alum is more potent in induction of cytokines production, it results in a relatively less robust humoral immune response compared to HBsAg/Montanide ISA 266 vaccine. This can be explained by liposome formation by Montanide ISA 266 that may result in better protection of HBsAg against protease enzymes. This protection than may lead to a higher transfer rate of native antigen from the injection site to the regional lymph nodes, leading to more prolonged exposure of B lymphocytes to the antigens with subsequent stronger humoral immune responses (21).

 Furthermore, IgG1 and IgG2a levels as markers for Th2 and Th1 patterns, respectively (31), were elevated more robustly by injection of HBsAg-Montanide 266 versus alum adjuvant vaccine. Studies are showing that Montanide, as an adjuvant, could promote class switching in the germinal center and antibody production, which is following the results of the present study (32). Similarly, Roohvand et al, demonstrated that HCV antigen, when was inoculated in combination with Montanide 720 plus CpG, is capable of increasing high titers of both IgG1 and IgG2a isotypes (19). Here, based on cytokines responses, alum was found to be more potent in the induction of the Th1 pattern. However, considering IgG1/IgG2a isotypes, it seems that Montanide adjuvant formulated vaccine is more influential in the induction of Th1/Th2 responses. Isotype switching is indeed under control of T cell cytokines, but this mechanism depends on a cytokine threshold. It means that switching may be triggered at a certain cytokine level and cytokines release over that threshold does not help induce a stronger humoral response. On the other hand, the increase of each isotype response may be related to antigen stability to stimulate B cells more efficiently. Thus, protection of the antigen against proteases and denaturing agent by Montanide adjuvant may be implicated for the achievement of superior IgG1/IgG2a responses (21, 33, 34).

 Overall, the results of the present study show that immunization with HBsAg formulated in Montanide ISA 266 adjuvant augments humoral immune responses compared to the HBsAg/alum vaccine. Application of another type of oil-based adjuvant not only may improve humoral immune response but also induce stronger cellular immune responses. Results of this study confirm that oil-based adjuvants can strengthen the humoral aspect of immune response compared to the commercially available HBsAg vaccine and formulation with a new oil-based adjuvant may stimulate other aspects of the immune system.
